# Deletion of Shp2 in bronchial epithelial cells impairs IL-25 production *in vitro*, but has minor influence on asthmatic inflammation *in vivo*

**DOI:** 10.1371/journal.pone.0177334

**Published:** 2017-05-08

**Authors:** Zhangwei Qiu, Jiesen Zhou, Fang Liu, Xuejun Qin, Yuanrong Dai, Yuehai Ke, Zhihua Chen, Wen Li, Songmin Ying, Huahao Shen

**Affiliations:** 1 Department of Respiratory and Critical Care Medicine, Second Affiliated Hospital, Zhejiang University School of Medicine, Hangzhou, Zhejiang, China; 2 Department of Pulmonary Medicine, Second Affiliated Hospital and Yuying Children’s Hospital, Wenzhou Medical University, Wenzhou, Zhejiang, China; 3 Department of Pathology and Pathophysiology, Program in Molecular Cell Biology and Institute of Respiratory Disease, Zhejiang University School of Medicine, Hangzhou, China; 4 State Key Lab for Respiratory Diseases, Guangzhou, China; Albert-Ludwigs-Universitat Freiburg, GERMANY

## Abstract

Shp2 played an important role in cigarette-smoke-mediated inflammation, surfactant homeostasis and asthmatic airway remodeling. However, whether shp2 plays a key role in epithelium-associated allergic reaction is still unknown. In this study, LPS and OVA were observed to induce the production of IL-25 in bronchial epithelial cells *in vitro* via the activation of MAPK p38 and JNK. Furthermore, blockage of Shp2 by its specific inhibitor PHPS1 or by siRNA-mediated depletion was found to reduce the production of IL-25 in epithelial cells as well as the up-regulated LPS-triggered activation of JNK but not p38. To confirm the role of intra-bronchial epithelial Shp2 in OVA-induced allergic reaction, we generated *CC10-rtTA/(tetO)7-Cre/Shp2*^*f/f*^ mice, where *Shp2* was conditionally knocked out in bronchial epithelial cells. Surprisingly, specific deletion of *Shp2* in bronchial epithelial cells showed a mild but insignificant effect on the expressions of epithelium-derived cytokines as well as TH2 and TH17 polarization following allergen-induced murine airway inflammation. Collectively, our data suggested that deletion of Shp2 impaired IL-25 production in bronchial epithelial cells *in vitro*, but might yet have minor influence on OVA-induced allergic reaction *in vivo*.

## Introduction

Asthma is a T-lymphocyte-controlled disease of the airway that is characterized with airway inflammation, overproduction of mucus and airway wall remodeling, leading to bronchial hyperreactivity and airflow obstruction[1]. The role of lung structural cells in asthma is poorly understood. The airway epithelium is more than just a structural barrier and has recently been considered as an essential immuno-regulator in asthmatic inflammation. The airway epithelium responds to inhaled allergens via pattern recognition receptors (PRRs), including Toll-like receptors (TLRs), Nod-like receptors (NLRs), C-type lectins and protease-activated receptors[2]. As is known, TLR4 triggering by LPS (Lipopolysacchatide) primes downstream cellular events, which are mediated by mitogen-activated protein kinase (MAPK) p38 and c-Jun N-terminal kinase (JNK), causing nuclear translocation of NF-κB. The latter then promotes the transcription of pro-inflammatory cytokine mRNA and release of cytokines[3]. PRRs that trigger on structural cells not only activate DCs but also innate lymphocytes and further polarize type 2 inflammation[4]. Lambrechts showed irradiated chimeric mice–in which Toll-like receptor 4 (TLR4) expressed on radio-resistant lung structural cells but not on dendritic cells (DCs)–were still primed type-2 responses to HDM (House dust mites). They also documented that deficiency of TLR4 on structural cells other than on DCs still inhibited HDM-induced allergic airway inflammation[5]. These findings suggest airway epithelial cells are necessary in driving allergic inflammation in the early stage of the disease.

TLR4 triggering on airway epithelial cells induces the production of innate pro-inflammatory cytokines, such as interleukin-25 (IL-25), interleukin-33 (IL-33), thymic stromal lymphopoietin (TSLP) and granulocyte-macrophage colony–stimulating factor (GM-CSF)[6]. These cytokines, named epithelium-derived cytokines, are related to the priming and the maintenance of type-2 responses, bridging innate immunity and adaptive immunity in the pathogenesis of asthma. It is demonstrated that IL-25 is expressed in epithelial cells, macrophages, microglia and CD8+ and CD4+T cells[7–10]. Although there is increasing knowledge about the function of IL-25 in asthma, the mechanism for how IL-25 is produced is still poorly studied. As is demonstrated, allergens, airborne pathogens and helminth could induce IL-25 production in lung epithelial cells[7,8,11,12]. However, the involved downstream regulators need further characterization. Although genomic sequence analysis of the upstream of the IL-25 encoding region suggested signal transducers and activators of transcription-6 (STAT-6), GATA-3 and Nuclear factor κB (NF-κB) binding sites might be involved, further functional investigation of these transcription factors have still not been reported[13]. This research aims to find the molecular signals involved in IL-25 production in airway epithelial cells.

Shp2 (Src homology 2 domain-containing phosphatase 2) is a member of intracellular classical protein tyrosine phosphatases (PTPs). Recently, Shp2 had been shown to have extensive function in a wide variety of diseases. Of interest, Shp2 is known to be universally expressed in the lung. It was reported that Shp2 took an important role in cigarette-smoke-mediated IL-8 overproduction in bronchial epithelial cells[14] and that loss of Shp2 in alveoli epithelia induced dysregulated surfactant homeostasis, resulting in spontaneous pulmonary fibrosis[15]. Our previous work revealed that Shp2 regulated TGF-β1 production in airway epithelia and asthmatic airway remodeling in mice[16]. However, whether Shp2 plays a role in epithelial inflammation of asthma is still unknown.

This present study will determine the role of Shp2 in the production of epithelium-derived cytokines and the value of intra-bronchial epithelial Shp2 in allergic reaction.

## Materials and methods

### Mice

Female C57BL/6 mice (wild-type, 6–8 week old) were purchased from the Animal Center of Zhejiang University (Hangzhou, Zhejiang, China) and housed in a conventional animal facility. Floxed Shp2 *(Shp2*^*f/f*^*)* mice (C57BL/6 background) were generous gifts from Dr. Gen-Sheng Feng (University of California at San Diego, USA)[17]. *CC10-rtTA*^*tg/-*^ and *(tetO)7CMV-Cre*^*tg/-*^ transgenic mice (C57BL/6 background) were obtained from Jackson Laboratories (Bar Harbor, ME). *CC10-rtTA/(tetO)*_*7*_*-Cre/Shp2*^*f/f*^ mice were generated by crossing *Shp2*^*f/f*^, *CC10-rtTA*^*tg/-*^ and *(tetO)*_*7*_*CMV-Cre*^*tg/-*^ transgenic mice. We obtained four kinds of phenotypes: *CC10-rtTA/(tetO)*_*7*_*-Cre/Shp2*^*f/f*^, *CC10-rtTA/Shp2*^*f/f*^, *(tetO)*_*7*_*-Cre/Shp2*^*f/f*^ *and Shp2*^*f/f*^. To detect the genotypes of mice, three pairs of primers, including *5'-AAA ATC TTG CCA GCT TTC CCC-3'* and *5'-ACT GCC CAT TGC CCA AAC AC-3'* for *CC10*, *5'-TGDDACGACCAAGTGACAGCAATG-3'* and *5'-AGAGACGGAAATCC ATCGCTCG-3'* for *(tetO)7CMV-Cre*, *5'-TGDDACGACCAAGTGACAGCAATG-3'* and *5'-AGAGACGGAAATCC ATCGCTCG-3’* for *Shp2*^*f/f*^. After exposure to DOX (Doxycycline, Sigma-Aldrich, USA) in drinking water for more than a week, intra-bronchial *Shp2* was specifically knocked out in *CC10-rtTA/(tetO)*_*7*_*-Cre/Shp2*^*f/f*^ mice, who were maintained in a pathogen-free animal center according to NIH guidelines. To detect Shp2-knockout allele, a forward primer (*5’-CAGTTGCAACTTTCT- TACCTC-3’*) in intron 3 and a reverse primer (*5’-GCAGGAGACTGCAGCTCAGTGATG-3’*) were designed. Besides ‘Genetic Controls’ (*CC10-rtTA/(tetO)*_*7*_*-Cre/Shp2*^*f/f*^ mice without DOX), we designed ‘Toxicity Controls’ (*CC10-rtTA/Shp2*^*f/f*^ mice with DOX) to exclude the toxicity of *rtTA*/DOX system[18]. An OVA-induced allergic model was established as described by Huahao Shen[1]. In brief, mice were sensitized by i.p. 20 μg chicken OVA (Grade V, Sigma-Aldrich, St Louis, MO, USA) and emulsified in 100 μl Imject Alum (Pierce, USA) on days 7 and 21. Control mice received the same volume of PBS (PBS controls) at each treatment time point. Subsequently, mice were challenged with an aerosol generated from 1% OVA or PBS for 40 minutes by ultrasonic nebulization (DeVilbiss, USA) from days 31 to 33. Mice sacrificing and bronchial alveolar lavage fluid (BALF) harvesting were performed as previously described[19]. In brief, inflammatory cells were obtained by cannulation of the trachea and lavage of the airway lumen with PBS. Cytospin slides were prepared by Wright-Giemsa staining. The analysis of cellular profiles in this study was blindly performed by the same investigator. This study was carried out in strict accordance with the recommendations in the Guide for the Care and Use of Laboratory Animals of the National Institutes of Health. The protocol was approved by the Committee on the Ethics of Animal Experiments at Zhejiang University. All surgery was performed under sodium pentobarbital anesthesia, and all efforts were made to minimize suffering.

### Lymphocyte isolation from the lung

After perfused with PBS, mice lungs were cut into small pieces and digested with collagenase type I (1 mg/ml, Sigma-Aldrich, US) and 150 μg/ml of DNase I (Sigma-Aldrich, US) at 37°C for 1 h, followed by grinding the tissues through a 40-μm cell strainer. Lymphocytes contained in the lungs were separated with Lympholyte^®^-M cell separation media (EZ-Sep^™^ Mouse 1X, Dakewe Bio, China) according to the manufacturer’s instruction.

### Preparation of lung homogenate

The frozen lung tissue samples were weighed and homogenized in RPMI 1640 medium (100 mg tissue per milliliter) on ice. The homogenates were centrifuged at 3000 × g for 15 minutes. The supernatants were collected for cytokine determination.

### Flow-cytometric analysis

Cells were stained with FITC-CD4, APC-IL-4 and PE-IL-17A (all from eBioscience, San Diego, CA, USA). For intracellular IL-17A staining, single-cell suspensions from the lung were stimulated for five hours with 10 ng/ml PMA (Phorbol myristate acetate, Multi Sciences, china), then 500 ng/ml ionomycin (Multi Sciences, china), and 10 μg/ml BFA (brefeldin A, Multi Sciences, china) was added during the final four hours of incubation. After stimulation, cells were harvested, followed by surface and intracellular staining according to the manufacturer’s instructions (eBioscience, San Diego, US). Stained leukocytes were washed and fixed in 2% paraformaldehyde and analyzed by flow cytometry using a FACSCalibur flow cytometer and CellQuest software (Becton Dickinson, Mountain View, CA, US). Positive staining thresholds were determined from appropriate isotype control staining.

### Cell culture

Beas-2b cells, a human bronchial epithelial cell line obtained from the Cell Bank, Chinese Academy of Sciences, were maintained in DMEM (Gibco, US) containing 10% FBS (Gibco, US) at 37°C in the presence of 5% CO2. MTECs were isolated from C57BL/6 mice using Pronase (sigma) and DNAse I (sigma, US) and plated (7.5 x 104–1.0 x105 cells per well) on collagen I-coated 12-transwell plate (Corning) as described by Lams[20]. Typical yields were 2.0 x 10^5^ cells/mouse. During the first 7–10 days, cells were cultured in proliferation media on the apical surface of the transwell polycarbonate membrane insert. When cells appear confluent and epithelial resistance reaches 1000 Ω/cm^2^, the cells differentiated to bronchial epithelial cells in differentiation media at air-liquid interface. The cells were allowed to differentiate for 10–14 days. LPS (L2630, Sigma-Aldrich, St Louis, MO, USA) was used to induce IL-25 production.

### Transfection of Shp2 siRNA

Beas-2bs were plated on 6-well plate of concentration of 2 x 10^5^ cells per well overnight to ~50% confluent. *Shp2*-specific siRNA (Santa Cruz, US) or scrambled siRNA (Santa Cruz, US) were transfected into Beas-2bs using GenMute^™^ (SignaGen Laboratories, US) according to the manufacturer’s instructions. Immunoblotting analysis was used to examine Shp2 silencing by siRNA 48 h after transfection.

### ELISA of cytokine levels

IL-25 Cytokine levels in BALF or cell culture medium supernatant were analyzed by ELISA using paired antibody (R&D Systems, Minnesota, US) following the manufacturer’s instructions.

### Quantitative RT-PCR (qRT-PCR)

Cells were lysed with TRIzol reagent (Invitrogen, US), and total RNA was extracted according to the manufacturer’s protocol. Reverse transcription was conducted using the PrimeScript RT reagent Kit (TaKaRa, Japan), and subsequent Real-time Quantitative PCR analysis was performed on an Applied Biosystems PRISM 7500 Sequence System using several primers (Table 1). Each sample was analyzed in triplicate.

**Table 1:** Primers for RT-PCR.

Gene	Forward	Reverse
Mouse β-actin	5’-AGAGGGAAATCGYGCGTGAC-3’	5’-CAATAGTGATGACCTGGCCGT-3’
Mouse IL-25	5’-TATGAGTTGGACAGGGACTTGA-3’	5’-TGGTAAAGTGGGACGGACTTG-3’
Mouse IL-33	5’-ATTTCCCCGGCAAAGTTCAG-3’	5’-AACGGAGTCTCATGCAGTAGA-3’
Mouse IL-4	5’-TTACCTTGACGGTGTTCATACAG-3’	5’-TCTGCTCCTATTCGACCACTATC-3’
Mouse IL-17A	5’-TCAGCGTGTCCAAACACTGAG-3’	5’-GACTTTGAGGTTGACCTTCACAT-3’
Mouse Foxp3	5’-CCC AGG AAA GAC AGC AAC CTT-3’	5’-TTC TCA CAA CCA GGC CAC TTG-3’
Human Gapdh	5’-AGGTCGGAGTCAACGGATTTG-3’	5’-CATGGGTGGAATCATATTGGAACA-3’
Human IL-25	5’-ATGTACCAGGTGGTTGCATTC-3’	5’-TGCTGTTGAGGGGTCCATCT-3’

### Immunoblotting analysis

Samples of protein (20ug) from the cell lysates were incubated with p-extracellular-signal-regulated kinases (ERK), p-p38, p-JNK, (Cell Signaling Technology, USA) and β-actin (Santa cruz, US) primary antibodies overnight. Immuno-reactive bands were visualized by a two-color infrared imaging system (Odyssey; LI-COR, Lincoln, NE).

### Data analysis

Results were presented as mean ± standard error of the mean (SEM). Data were analyzed with Student’s t-tests (two-tailed) or one-way ANOVA. Differences were considered statistically significant if *P*<0.05.

## Results

### Ovalbumin or lipopolysaccharide elicited production of IL-25

**Fig 1:**
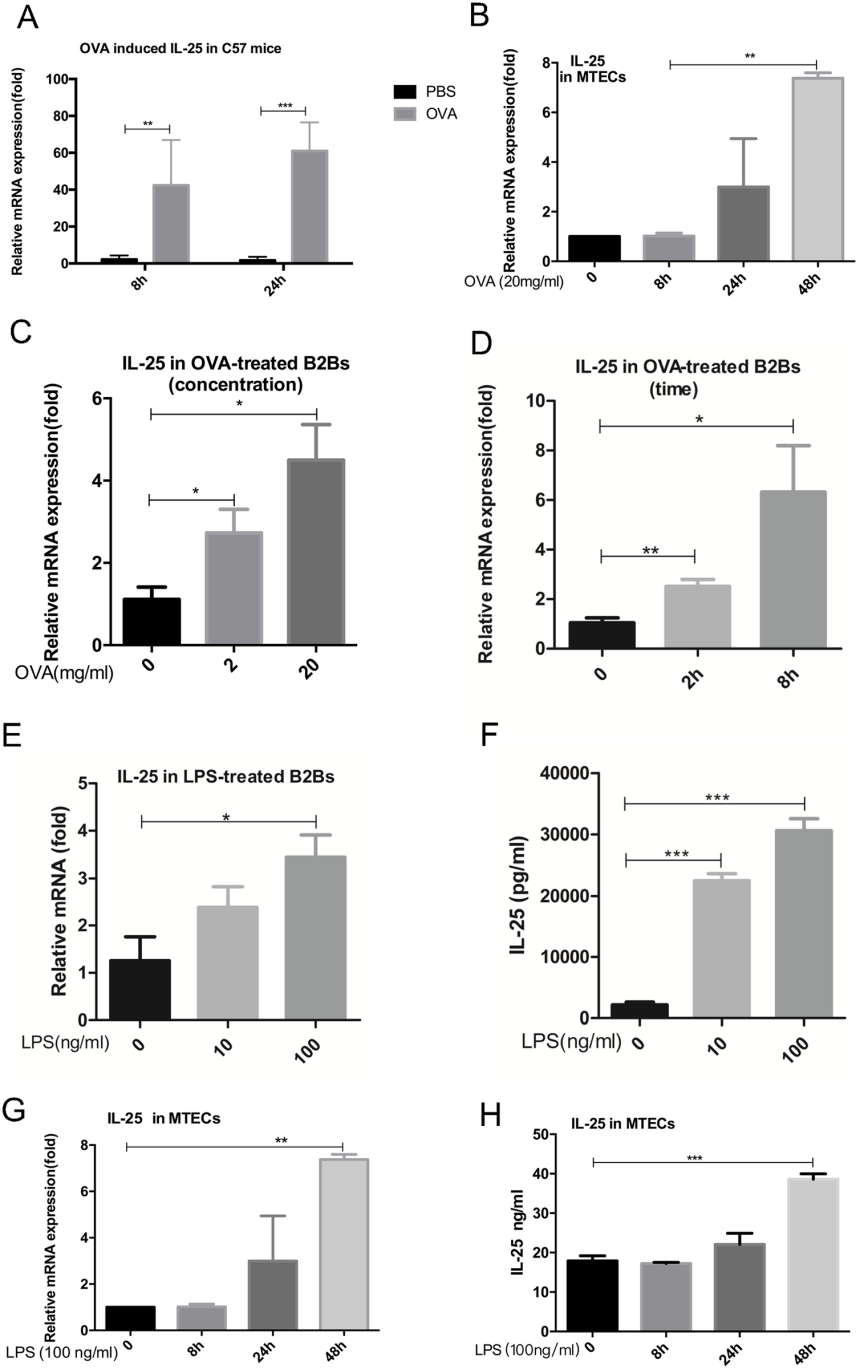
OVA- or LPS-elicited production of IL-25. (A) C57BL/6 mice were sensitized and challenged with OVA, and IL-25 mRNA in lung tissue was measured by q-PCR. (B) MTECs were treated with 20 mg/ml OVA for different periods of time (0, 8, 24 and 48 hours), followed by detection of IL-25 mRNA level. Beas-2b cells were treated with OVA of different concentrations (2 and 20 mg/ml) for 8 hours (C) or 20 mg/ml OVA for different periods of time (0, 2, 8 hours) (D), followed by detection of IL-25 mRNA. Serum-free Beas-2bs were stimulated by LPS of different concentrations (0 ng/ml, 10 ng/ml, 100 ng/ml). IL-25 mRNA (E) were detected by q-PCR, IL-25 protein released into the supernatant (F) were measured by ELISA. MTECs were treated with 100 ng/ml LPS for different periods of time (0, 8, 24 and 48 hours), IL-25 mRNA (G) were detected by q-PCR, IL-25 protein released into the supernatant (H) were measured by ELISA. Results were expressed as mean ± SEM of three independent experiments. B2Bs: Beas-2b cells. *p <0.05, **p<0.01 ***p<0.001. B2Bs: Beas-2b cells.

EDCs can be induced by allergens, such as OVA (ovalbumin)[21], HDM[5], Alternaria, Aspergillus oryzae and ragweed[22]. To confirm the previous finding, we measured the level of IL-25 mRNA in lung tissues of OVA-treated C57/BL6 mice (Fig 1A). As is known, IL-25 is one of epithelium-derived cytokines. Therefore, we detected IL-25 mRNA expression in OVA-treated primary mouse tracheal epithelial cells (MTECs), which were separated from C57BL/6 mice, and Beas-2b cells. As is shown, in Beas-2b cells, the level of IL-25 mRNA 48 hours after OVA treatment were significantly elevated in MTECs (Fig 1B); in Beas-2b cells, the results showed that IL-25 could be triggered by OVA in a concentration-dependent manner (Fig 1C) as well as a time-dependent manner (Fig 1D). Since OVA cannot be recognized by PRRs on the epithelial membrane, we assumed that the contaminated LPS activated epithelium. Serum-free Beas-2b cells were treated with LPS of different concentrations. As a result, when stimulated by LPS, the level of IL-25 mRNA in Beas-2b cells markedly increased (Fig 1E), and the concentration of IL-25 in the culture supernatant were significantly elevated in a concentration-dependent manner as well (Fig 1F). To further illustrate this finding, MTECs were treated with 100 ng/ml LPS. Similarly, the level of IL-25 mRNA significantly increased in LPS-treated MTECs (Fig 1G), and the concentration of IL-25 protein in the culture supernatant of LPS-treated MTECs were significantly augmented (Fig 1H).

### LPS induced IL-25 via the activation of MAPK p38 and JNK

**Fig 2:**
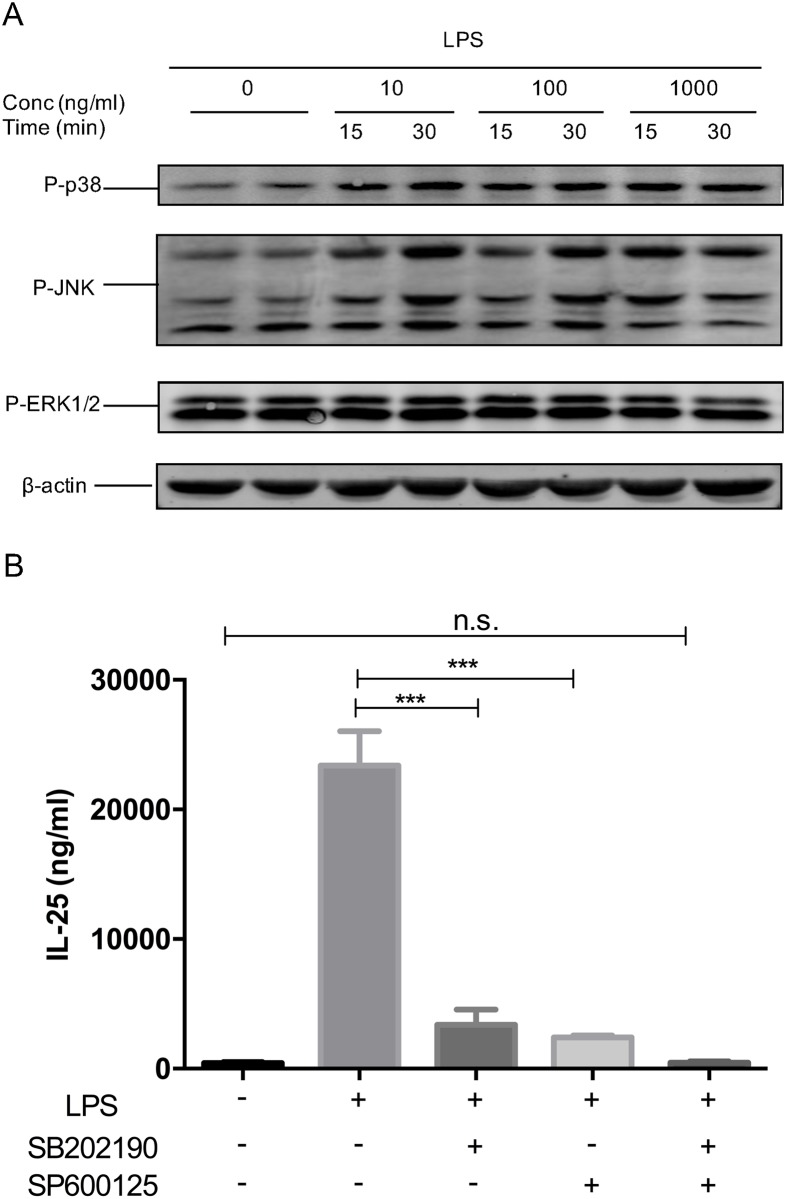
LPS-induced IL-25 via the activation of MAPK p38 and JNK. (A) Serum-free Beas-2bs were treated with different concentrations (10, 100 and 1000 ng/ml) of LPS for different periods of time (15 and 30 min). Phosphorylation levels of p38 and JNK were measured via immunoblotting. (B) Beas-2bs were pre-treated with SB202190 and/or SP600125 30 min, followed by stimulation of LPS. IL-25 protein concentration of the cell culture supernatant was measured in the cell culture 8 hours after giving LPS. Results were expressed as mean ± SEM of three independent experiments. ***p<0.001, ^n.s.^*p>0*.*05*.

Activation of TLR4 by LPS results in downstream cellular events that are mediated by MAPK p38 and JNK. To confirm that, serum-free Beas-2b cells were treated with different concentrations (0 ng/ml, 10 ng/ml, 100 ng/ml, 1000 ng/ml) of LPS for different periods of time (15 minutes, 30 minutes). Phosphorylation levels of p38 and JNK were measured via immunoblotting. The results showed that LPS was powerful to activate MAPK p38 and JNK (Fig 2A). Based on that, we wonder whether IL-25 production by epithelial cells depends on MAPK p38 and JNK. Therefore, serum-free Beas-2b cells were pre-treated with p38 inhibitor SB202190 (32 nM) and/or JNK inhibitor SP600125 (40 nM) for 30 minutes, followed by LPS stimulation. The IL-25 protein concentration of the cell culture supernatant was measured 8 hours after receiving LPS. We found that both SB202190 or SP600125 efficiently inhibited LPS-induced IL-25 in Beas-2bs individually, and their combination nearly totally inhibited its production (Fig 2B). Therefore, we proved that LPS induced IL-25 via activation of MAPK p38 and JNK.

### Blockage of Shp2 down-regulated LPS-triggered activation of MAPK JNK

**Fig 3:**
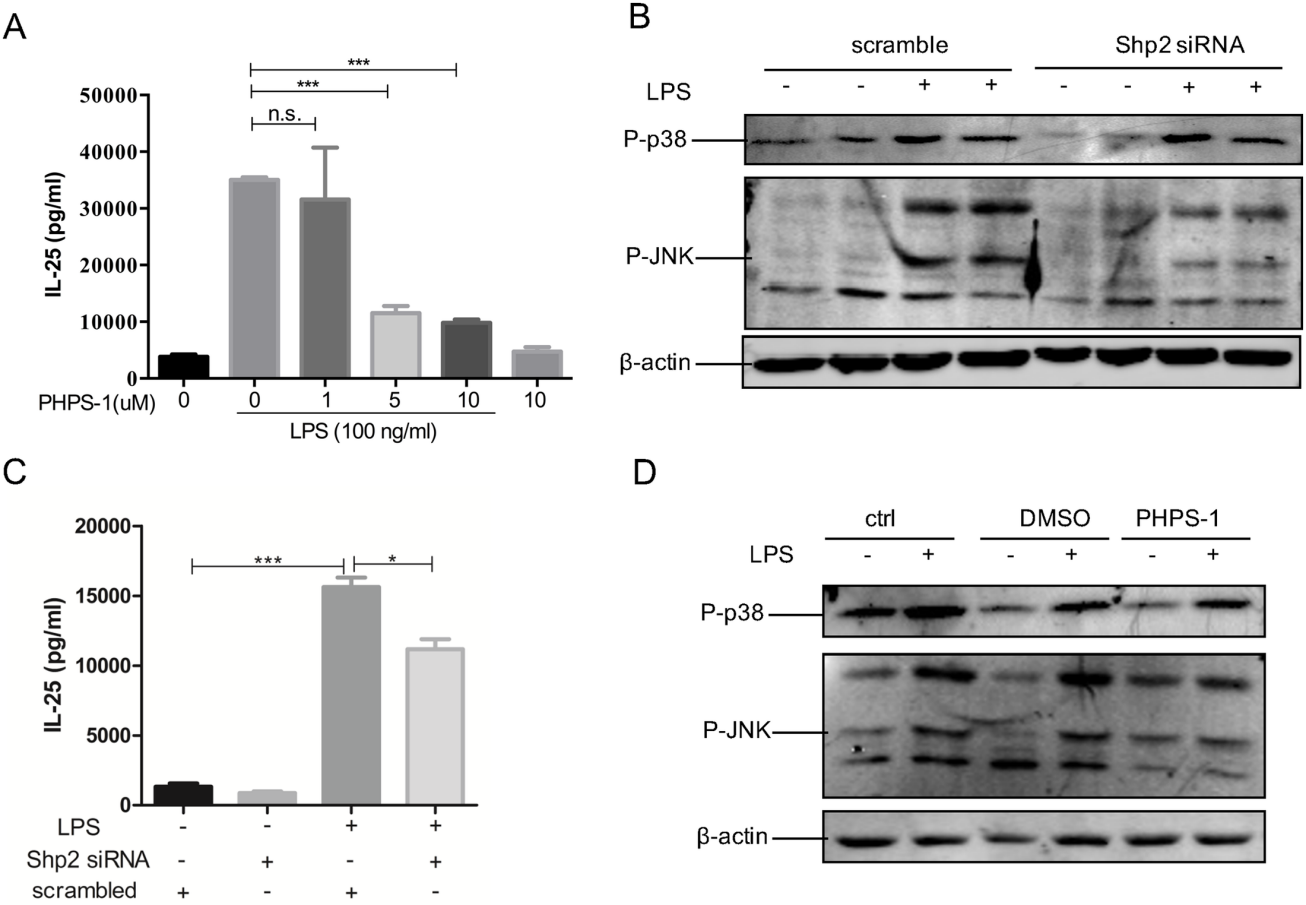
Blockage of Shp2 down-regulated LPS-triggered activation of MAPK JNK. Beas-2b cells were pretreated with PHPS-1 for 15 min, followed by LPS treatment. (A) The protein concentration of IL-25 was measured in 8 hours in the culture media supernatant. (B) Cell total protein was extracted 30 min after LPS stimulation to detect the expression of P-p38 and P-JNK. (C) *Shp2* siRNA was transfected into Beas-2bs, followed by LPS treatment 40 hours later. Eight hours after LPS treatment, the protein concentration of IL-25 was measured in the culture media supernatant. (D) Cell total protein was extracted 30 min after LPS stimulation to detect the expression of P-p38 and P-JNK. Results were expressed as mean ± SEM of three independent experiments. *p<0.05, ***p<0.001, ^n.s.^*p*>0.05. DMSO: Dimethyl Sulphoxide, used to dissolve PHPS1.

To explore the role of Shp2 in regulating the secretion of IL-25 in bronchial epithelial cells, Shp2 inhibitor PHPS-1 and *Shp2* siRNA were used. Pretreated Beas-2b cells with PHPS-1 before LPS stimulation led to significant inhibition of the secretion of IL-25 in a concentration-dependent manner (Fig 3A). Meanwhile, when we transfected Shp2 siRNA into Beas-2bs, LPS-induced IL-25 was also significantly repressed (Fig 3C). Based on those findings, we concluded that Shp2 promoted the production of IL-25 in epithelial cells. Since it was verified that LPS induced IL-25 selectively via p38 and JNK, we wondered if Shp2 promoted LPS induced IL-25 via these signals as well. First of all, whether Shp2 regulates MAPK p38 and JNK should be verified. The Shp2-specific inhibitor PHPS-1 was used. Serum free Beas-2b cells were pre-treated with 5 uM PHPS-1, and we found PHPS-1 significantly inhibited LPS-activated JNK but not p38. Beas-2b cells were transfected by *Shp2* siRNA, followed by LPS (100 ng/ml) stimulation. Results showed that *Shp2* siRNA inhibited LPS induced phosphorylation of JNK but not that of p38 as well (Fig 3D). We conclude that Shp2 selectively regulates LPS-triggered activation of MAPK JNK (except for p38).

### Generation of transgenic mice and groups

**Fig 4:**
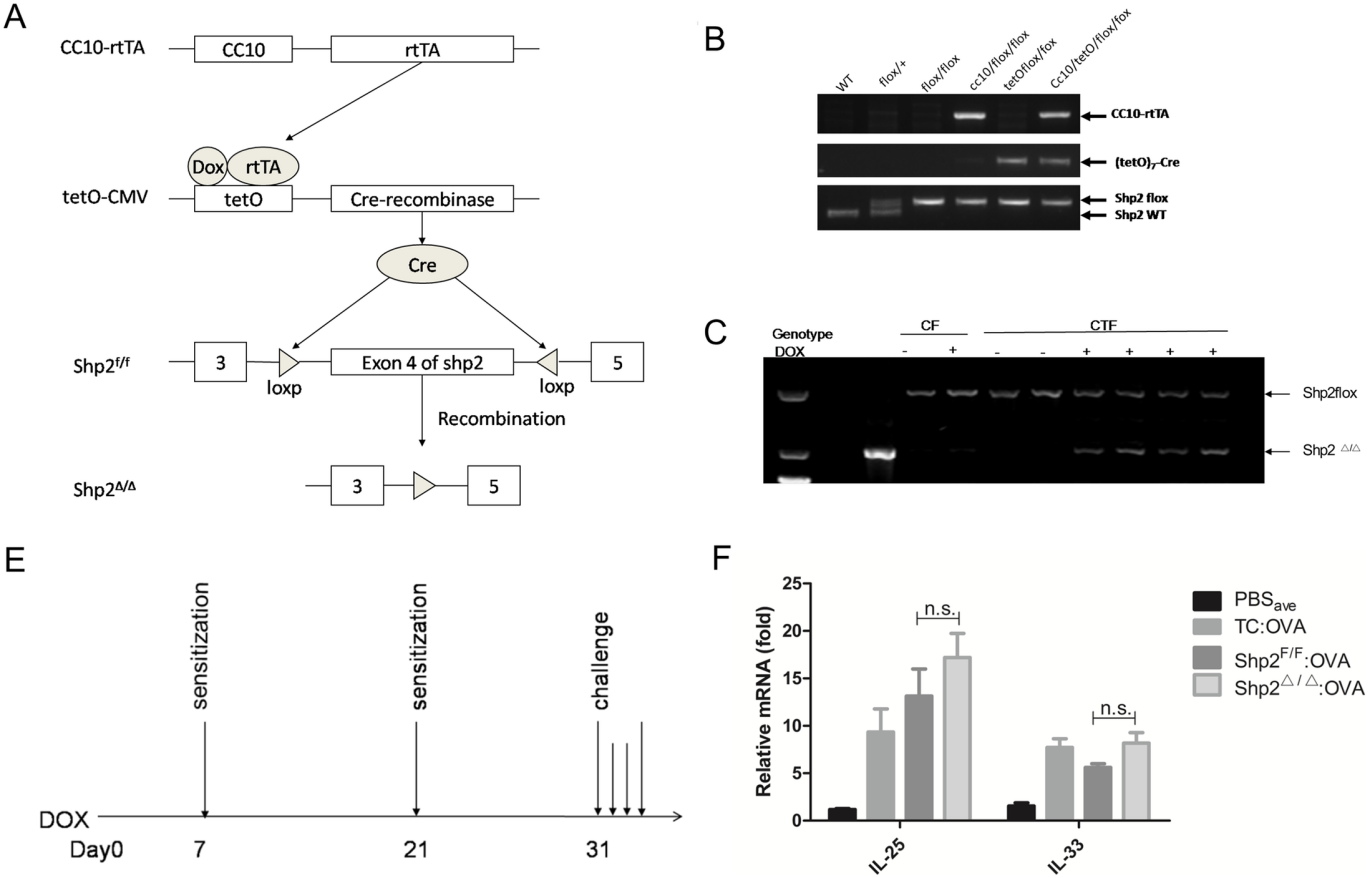
Knockout of *Shp2* in bronchial epithelial cells have a mild effect on epithelium-derived cytokine production *in vivo*. (A) Schematic map of the generation of triple-transgenic mice *CC10-rtTA/(tetO)7-Cre/Shp2*^*f/f*^. The human *CC10* promoter was used to express the reverse tetracycline transactivator (*rtTA*) in clara cells. In the presence of DOX, *rtTA* binds to the *tetO-CMV* promoter, activating the transcription of Cre-recombinase, removing the floxed exon 4 of the *Shp2* gene, resulting in specific deletion of *shp2* in Clara cells. (B) Genotyping was performed by PCR assays using mouse tail genomic DNA. (C) Inactivation of shp2 allele was confirmed by PCR of genomic DNA isolated from lung tissues of *CC10-rtTA/(tetO)7-Cre/Shp2*^*f/f*^ mice after 7-day treatment with DOX (through drinking, 2 mg/ml in H_2_O) or H_2_O (as control). (D) OVA-induced allergic asthmatic mouse model. Five-to-seven-week animals were given drinking water with or without DOX (2mg/ml) from Day 0, then they were sensitized with intra-peritoneal injection of OVA (80ug/mice) or PBS on Day 7 and Day 21. Three consecutive challenges of OVA aerosol (1%) once a day for 3 continual days from Day 32 to Day 34 were performed; the mice were then sacrificed. (E) The levels of IL-25 and IL-33 mRNA in lung tissues were detected by RT-PCR. Results were expressed as mean ± SEM of three independent experiments. ^n.s.^*p*>0.05. CF: *CC10-rtTA/(tetO)*_*7*_*-Cre/Shp2*^*f/f*^, CTF: *CC10-rtTA/Shp2*^*f/f*^. PBS_ave_: average value of three PBS subgroups, including *CC10-rtTA /Shp2*^*f/f*^:DOX (toxicity control, TC), *CC10-rtTA/(tetO)7-Cre/Shp2*^*f/f*^:H_2_O (Shp2^F/F^) and *CC10-rtTA/(tetO)7-Cre/Shp2*^*f/f*^:DOX (Shp2^△/△^).

Shp2 is universally highly expressed in lungs of healthy mice (S3A Fig). Immunoblotting showed that Shp2 is slightly increased, but not significantly, in isolated bronchial epithelial cells of OVA-treated allergic mice (S3C Fig); this is consistent with our previous finding[16]. As is shown, background shp2 is so high, however, that we did not find a significant increase in the whole lung of allergic mice (S3A and S3B Fig). To clarify the role of Shp2 in an allergic mouse model, we generated *CC10-rtTA/(tetO)7-Cre/Shp2*^*f/f*^ mice by crossing floxed *Shp2* (*Shp2*^*f/f*^) with mice carrying the human *CC10* promoter-*rtTA* and *(tetO)7-CMV*-Cre transgenes (*CC10-rtTA/(tetO)7-Cre*). We obtained four kinds of phenotypes: *CC10-rtTA/(tetO)*_*7*_*-Cre/Shp2*^*f/f*^, *CC10-rtTA/Shp2*^*f/f*^, *(tetO)*_*7*_*-Cre/Shp2*^*f/f*^ and *Shp2*^*f/f*^. Triple transgenic mice *CC10-rtTA/(tetO)*_*7*_*-Cre/Shp2*^*f/f*^ and littermate *CC10-rtTA/Shp2*^*f/f*^ control mice were used for the experiments. When given doxycycline in drinking water, *CC10-rtTA/(tetO)7-Cre/Shp2*^*f/f*^ mice expressed cre remonbinase in their bronchial epithelia cells to recognize the *loxp/loxp* sequence, leading to subsequent *Shp2* inactivation (Fig 4A). For research use, we mated male *CC10-rtTA/Shp2*^*f/f*^ mice with female *CC10-rtTA/(tetO)7-Cre/Shp2*^*f/f*^ mice. Then we obtained four genotypes as filial generation: *Shp2*^*f/f*^, *CC10-rtTA/Shp2*^*f/f*^, *(tetO)7-Cre/Shp2*^*f/f*^, and *CC10-rtTA/(tetO)7-Cre/Shp2*^*f/f*^ (Fig 4B). Genomic DNA analysis of the lungs of *CC10-rtTA/(tetO)7-Cre/Shp2*^*f/f*^ mice showed that the *Shp2* gene was disrupted when 2mg/ml DOX was administered to the mice for seven consecutive days (Fig 4C). Since we had difficulty in double immunofluorescence labeling of CC10 and Shp2, we analyzed the Shp2 allele of genomic DNA isolated from the brain and liver of *CC10-rtTA/(tetO)7-Cre/Shp2*^*f/f*^ mice after DOX exposure, and we found that Shp2^Δ/Δ^ was not detectable in these organs (S4 Fig). Therefore, we successfully generated an inducible bronchial epithelia-specific *Shp2* knockout mouse model. *rtTA*/DOX toxicity in *SP-C-rtTA* mice was reported by Morimoto and Kopans[18], suggesting additional controls should be designed in the experiments of these strains, and proper controls must include paralleled molecular analysis of DOX-fed *rtTA* strains in matched background to their bi- and tri-transgenic littermates. In order to avoid possible bias caused by *rtTA*/DOX toxicity, in our investigation, both OVA mice and PBS controls included three subgroups: *CC10-rtTA /Shp2*^*f/f*^:DOX (toxicity control, TC), *CC10-rtTA/(tetO)7-Cre/Shp2*^*f/f*^:H_2_O (Shp2^F/F^) and *CC10-rtTA/(tetO)7-Cre/Shp2*^*f/f*^:DOX (Shp2^△/△^).

### Knockout of Shp2 in bronchial epithelial cells have a mild effect on epithelium-derived cytokine production *in vivo*

To further determine whether intra-epithelial Shp2 functioned on OVA-induced airway inflammation, we examined the levels of epithelium-derived cytokines IL-25 and IL-33 in the lung tissue of OVA-sensitized Shp2-deficient or control mice 24 hours after OVA inhalation. The results showed that both cytokines were elevated in OVA-treated mice compared to PBS control. However, in the OVA groups, the levels of both cytokines in Shp2-deficient mice were insignificantly different from those of shp2 ^F/F^ mice (Fig 4E).

### Intra-bronchial epithelial Shp2 depletion exert little effect on OVA-induced inflammation

**Fig 5:**
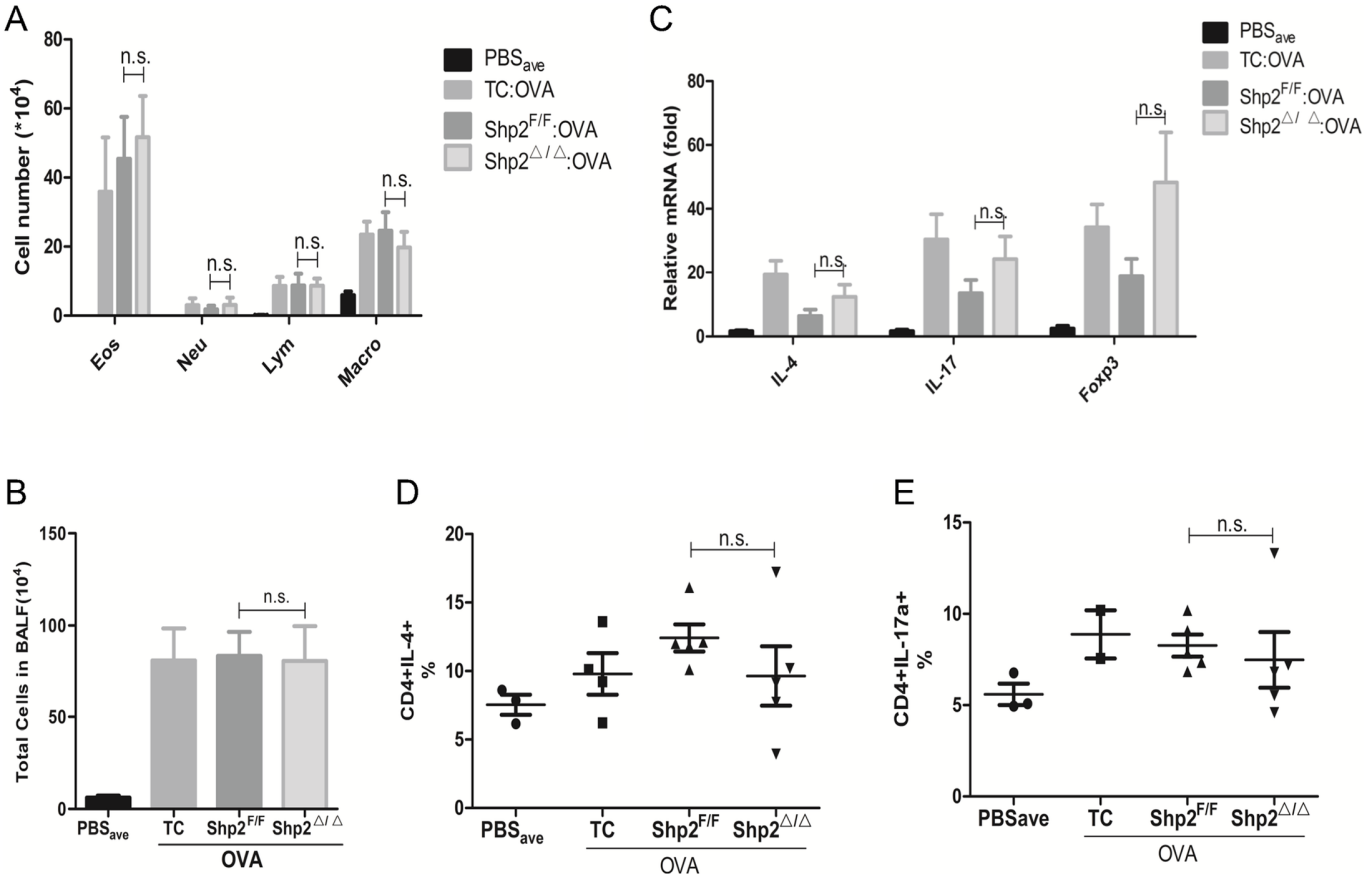
Intra-bronchial epithelial Shp2 depletion exert little effects on OVA-induced inflammation and TH2 and TH17 polarization. Twenty-four hours after the last challenge, the cellular profiles (A) were analyzed, and total cell numbers (B) were counted in BALF of mice. (C) IL-4, IL-17 and foxp3 mRNA were measured in the lung tissues of the mice. (D) Ratios of TH2 (stained by FITC-CD4 and APC-IL-4) and TH17 (stained by FITC-CD4 and PE-IL-17A) in the lung homogenates. (n = 5 mice/group, in two separate experiments). Results were expressed as mean ± SEM. ^n.s.^*p* >0.05. PBS_ave_: average value of three PBS subgroups, including *CC10-rtTA /Shp2*^*f/f*^:DOX (toxicity control, TC), *CC10-rtTA/(tetO)7-Cre/Shp2*^*f/f*^:H_2_O (Shp2^F/F^), and *CC10-rtTA/(tetO)7-Cre/Shp2*^*f/f*^:DOX (Shp2^△/△^). ^n.s.^*p>0*.*05*. Eos: Eosinophil, Neu: Neutrophil, Lym: Lymphocyte, Macro: Macrophage.

As previously described, inflammatory cells infiltrating in BALF of mice were assayed 24 hours after the last OVA inhalation. We found that the OVA exposure elevated the total cellular number and increased the total eosinophils, as was previously shown. However, both the total cell number and the cellular profile of OVA-treated Shp2^△/△^ mice showed insignificant differences from OVA-treated Toxicity Control (TC) and Genetic Control (Shp2^F/F^) (Fig 5A and 5B) mice. Thus, we demonstrated that intra-bronchial epithelial Shp2 depletion exerts a small effect on OVA-induced inflammatory cell infiltration in BALF of mice.

### Knockout of Shp2 in bronchial epithelial cells had no effects on TH2 and TH17 polarization

As is known, TH2 response is one of the characteristics of allergic asthma. Our findings showed that TH cytokines, including IL-4, IL-17 and Foxp3, in the lung tissue of OVA-sensitized Shp2-deficient mice 24 hours after OVA inhalation were not significantly changed compared to Toxicity Control (TC) and Genetic Control (Shp2^F/F^) mice (Fig 5C). To further observe whether the deficiency of Shp2 affected the polarization of asthma-related TH subsets, we detected the ratios of TH2 (stained by FITC-CD4 and APC-IL-4) and TH17 (stained by FITC-CD4 and PE-IL-17A) in the lung tissue suspension. We found the ratios of both TH2 and TH17 among three OVA-given groups were almost equal (Fig 5D and 5E).

## Discussion

In the present study, our data indicated that LPS-contaminated OVA activated MAPK p38 and JNK, promoting IL-25 production in bronchial cells. We also found that blockage of Shp2 by its specific inhibitor PHPS1 or by siRNA-mediated depletion selectively down-regulated LPS-triggered activation of JNK rather than p38. We still determined that inhibition of Shp2 by its specific inhibitor PHPS-1 resulted in the reduction of IL-25 production in epithelial cells *in vivo*. However, specific deletion of Shp2 in bronchial epithelial cells showed a mild but insignificant effect on the expressions of epithelium-derived cytokines as well as TH2 and helper TH17 polarization following allergen-induced murine airway inflammation. Collectively, our data suggest that Shp2 promote IL-25 production in bronchial epithelial cells via JNK activity *in vitro* but might yet have little influence in OVA-induced allergic airway inflammation *in vivo*.

We have obtained some data regarding p38 and JNK for OVA *in vitro* (S1 Fig), and they show the same pattern as LPS. However, that result is not reproducible due to the indefinite concentrations of LPS contained in OVA among the different lots. That is why we chose LPS alone to further explore the role of Shp2 in IL-25 production. LPS-free Ova was not used for some consideration. There are some experimental evidences that support that LPS modulates an allergen-induced host response though activation of DC and epithelial cells via TLR4 signaling[5]. Epithelial TLR4 recognizes and responds to PAMPs (e.g., HDM and LPS) but not LPS-free OVA[2], and that is why our experiments are conducted with OVA *in vivo* and LPS *in vitro*.

Generally, IL-25 mRNA/proteins are universally expressed both in lung epithelial cells and hematopoietic cell lineages, such as T cells, mast cells[10,23,24], alveolar macrophages[8], eosinophils and basophils[25]. In airway inflammatory diseases, IL-25 can be induced by inhaled stimulators. The downstream regulators involved in the production of IL-25 were still not clear. It is acknowledged that triggering of TLR4 by LPS activates MAPK p38 and JNK, resulting in nuclear trans-location of NF-κB in response to inhaled stimulator, leading to proinflammatory cytokine mRNA transcription. However, how epithelium-derived cytokine IL-25 is induced by LPS has not been clearly deciphered. Genomic sequence analysis of the upstream of the IL-25 encoding region suggested STAT6, GATA-3 and NF-κB binding sites might be involved[3], but functional investigation of these transcription factors have still not been reported[26]. GATA3 plays a key role in Th polarization in asthma, and it has been indicated previously that there is no significant difference in the expression of GATA-3 protein in bronchial biopsies of normal subjects compared with asthmatic patients[27], we speculate that GATA3 might not be involved in asthma-related cytokines production in epithelial cells. We have already tried to investigate the role of stat6 in IL-25 production. Our results showed that IL-13, but not LPS, activates stat6 in bronchial epithelial cells. Further, although IL-13 was powerful to activate stat6, it was incapable of inducing IL-25 production in bronchial epithelial cells. We also demonstrated that shp2 was not involved in IL-13-activated stat6 (S2 Fig). Our data proves that LPS induces IL-25 dependent of MAPK p38 and JNK.

Kinases and phosphatases are respectively responsible for two reciprocal molecular events: phosphorylation and de-phosphorylation. Protein tyrosine phosphatase Shp2 is composed of two SH2 domains on the N-terminal and C-terminal PT domain. These two different domains have different functions, making Shp2 exhibit reciprocal regulatory roles according to different cellular localization and different stimuli[28]. In this study, we suggest that Shp2 positively regulates LPS-activated MAPK JNK and subsequently up-regulates IL-25 production in bronchial epithelial cells. Shp2 activation of the tyrosine phosphatase domain leads to subsequent de-phosphorylation of a series of substrates. Although several molecular mediators–such as apoptosis signal-regulating kinase-1 (ASK1)[29], epidermal growth factor receptor (EGFR) (Tyr992)[30], Grb2-associated binder-1 (Gab1)[31], Ras GTPase-activating protein (RasGAP) (Tyr771)[32], phosphoprotein associated with glycosphingolipid-enriched membrane microdomains (PAG)/ CSK-binding protein (Cbp), paxillin[33,34], etc.–have been confirmed as substrates of Shp2, that is, those mediators are not involved in the LPS-triggered signaling pathway. As is reported, TAK1 (transforming growth factor-β activated kinase-1) is substantial to activate the NF-κB- and JNK-dependent signaling pathway in innate immune response[35–38]. Several different phosphatases have been demonstrated to dephosphorylate Thr187 and to bind TAK1, suppressing TAK1 activity[39–41]. Base on these evidences and our novel findings, we believe that in LPS-stimulating bronchial epithelial cells, Shp2 activates the JNK signaling pathway via the de-phosphorylation of TAK1.

Along with our partners, we previously documented that Shp2 played an important role in acute cigarette-smoke-mediated IL-8 overproduction[14], loss of Shp2 in alveoli epithelia induced deregulated surfactant homeostasis, resulting in spontaneous pulmonary fibrosis[15], and Shp2 regulated TGF-β1 production in airway epithelia and asthmatic airway remodeling in mice[16]. Here, we further studied the impact of Shp2 on the expression of epithelium-derived cytokine IL-25, which was important in type 2 immune response in asthma. We generated an inducible bronchial epithelia-specific *Shp2* knockout mouse: *CC10-rtTA/(tetO)7-Cre/Shp2*^*f/f*^ mice. Surprisingly, animal experiments showed specific knockout of *Shp2* in bronchial epithelial cells have little effect on epithelium-related cytokine production in vivo. In other words, there are differences between data *in vitro* and *in vivo*. These differences are unlikely due to species differences in gene regulation between humans and mice, since we documented increased IL-25 in both the human bronchial cell line and primary mouse tracheal epithelial cells. Therefore, two points may aid understanding. Firstly, our previous work showed PHPS-1 alleviated eosinophilic airway inflammation and airway hyper-responsiveness as well as eosinophils in allergic wild-type mice, suggesting Shp2 is indeed involved in asthma pathogenesis. This present research aims to clarify whether intraepithelial Shp2 or non-epithelium-derived Shp2 play a role. Also, the findings suggest that not intraepithelial Shp2 but non-epithelium-derived Shp2 might be involved in airway inflammation[42]. Secondly, IL-25 was not only expressed in epithelial cells but also in other inflammatory cells, such as macrophages, microglia, and CD8+ and CD4+T cells. Inhibition of epithelial IL-25 production by specifically Shp2 deletion might be compensated by other cell sources. Generally, IL-25 mRNA/proteins are universally expressed both in airway epithelial cells and hematopoietic cell lineages, such as T cells, mast cells[10,23,24], alveolar macrophages[8], eosinophils and basophils[25]. By adoptive transfer of either T cells, mast cells or bone marrow cells from IL-25-deficient mice, Suzukawa et al. found that IL-25 produced by epithelial cells was indispensable for induction of Th2-type/eosinophilic airway inflammation[43]. Our data points to a complementary possibility that epithelial-cell-derived IL-25, which is independently substantial in priming TH2-type/eosinophilic airway inflammation and is insufficient to further maintain and amplify the inflammation. Instead, we supposed TH2-polarized T cells might be the main source of IL-25 to sustain the inflammation and influence the severity of asthma. Our data also suggest non-epithelium-derived Shp2 play a key role in allergic airway inflammation other than intra-epithelial Shp2.

## Supporting information

S1 Fig OVA activates MAPK p38 and JNK in Beas-2b cells. Serum-free Beas-2bs were treated with different concentrations (0.2, 2 and 20mg/ml) of OVA for different periods of time (15, 30 and 60 min). Phosphorylation levels of ERK, p38 and JNK were measured via immunoblotting.
(TIF)

S2 Fig Shp2 exerts no effect on IL-13-activated stat6; IL-13 alone is insufficient to induce IL-25 in bronchial epithelial cells. (A) Serum-free Beas-2bs were treated with 20 ng/ml IL-13 for different periods of time (15, 30 and 60 min). Phosphorylation levels of stat6 were measured via immunoblotting. (B) Beas-2bs were treated with 20 ng/ml IL-13 or 100 ng/ml LPS for 8 hours, supernatants were harvested, and IL-25 concentrations were measured through Elisa. (C) Shp2 siRNA were transfected into Beas-2bs, followed by LPS treatment 48 hours later. Cell total protein was extracted 30 min after LPS stimulation to detect the expression of P-stat6. ***p<0.001, ^n.s.^*p>0*.*05*.
(TIF)

S3 Fig Shp2 is universally expressed in the lung; it is especially highly expressed in bronchial epithelial cells and was slightly elevated in allergic bronchial epithelial cells. (A) The cellular distribution of Shp2 (brown staining cells) in the lungs of healthy and allergic mice by immunohistology. (B) Total Shp2 protein expression in the lungs of healthy and allergic mice by immunoblotting. (C) Shp2 protein expression in MTECs that respectively isolated from healthy and allergic mice (immunoblotting).
(TIF)

S4 Fig Shp2^Δ/Δ^ was undetectable in genomic DNA isolated from other organs. Genomic DNA was isolated from the brain and liver of *CC10-rtTA/(tetO)7-Cre/Shp2*^*f/f*^ mice after 7-day treatment with DOX (through drinking, 2 mg/ml in H_2_O) and Shp2 allele was detected by PCR.
(TIF)
