# Supplementary figures and images for: Deletion of Shp2 in bronchial epithelial cells impairs IL-25 production in vitro, but has minor influence on asthmatic inflammation in vivo
Source: PLoS One. 2017 May 8;12(5):e0177334. doi: 10.1371/journal.pone.0177334 (PMC5421800; doi:10.1371/journal.pone.0177334)

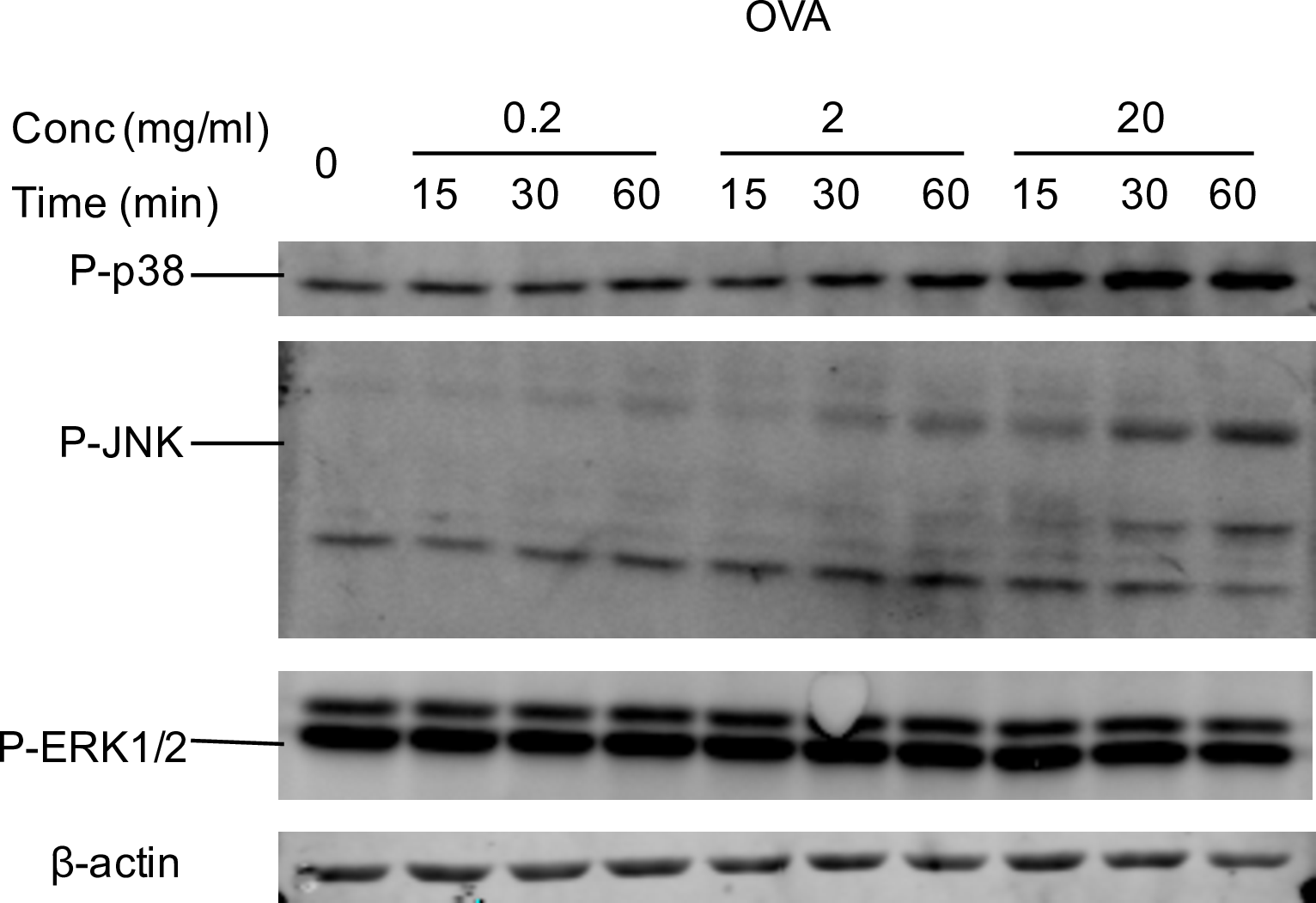

Supplement: S1 Fig — Serum-free Beas-2bs were treated with different concentrations (0.2, 2 and 20mg/ml) of OVA for different periods of time (15, 30 and 60 min). Phosphorylation levels of ERK, p38 and JNK were measured via immunoblotting. (TIF) [file pone.0177334.s001.tif]

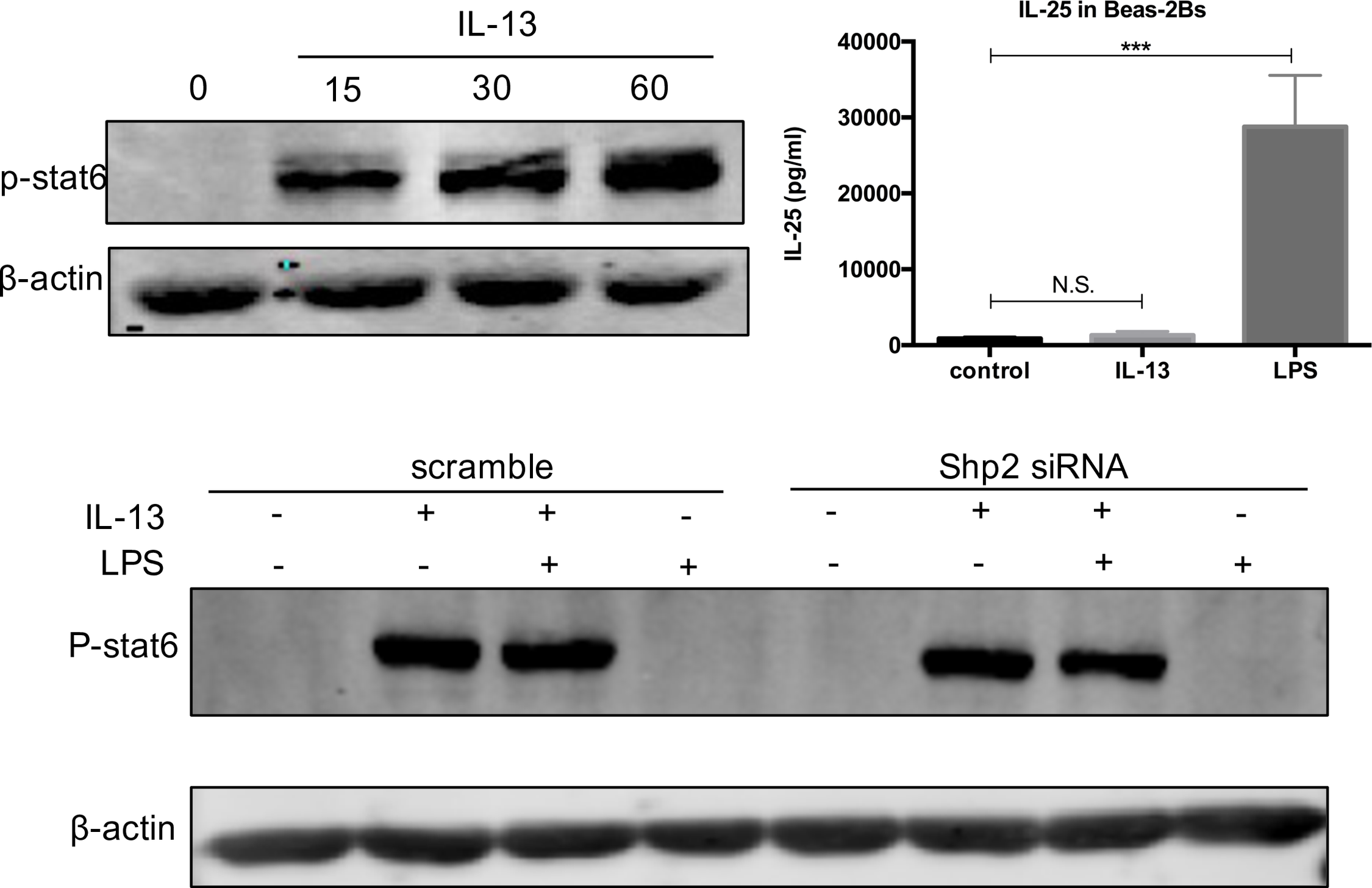

Supplement: S2 Fig — (A) Serum-free Beas-2bs were treated with 20 ng/ml IL-13 for different periods of time (15, 30 and 60 min). Phosphorylation levels of stat6 were measured via immunoblotting. (B) Beas-2bs were treated with 20 ng/ml IL-13 or 100 ng/ml LPS for 8 hours, supernatants were harvested, and IL-25 concentrations were measured through Elisa. (C) Shp2 siRNA were transfected into Beas-2bs, followed by LPS treatment 48 hours later. Cell total protein was extracted 30 min after LPS stimulation to detect the expression of P-stat6. ***p<0.001, n.s.p>0.05. (TIF) [file pone.0177334.s002.tif]

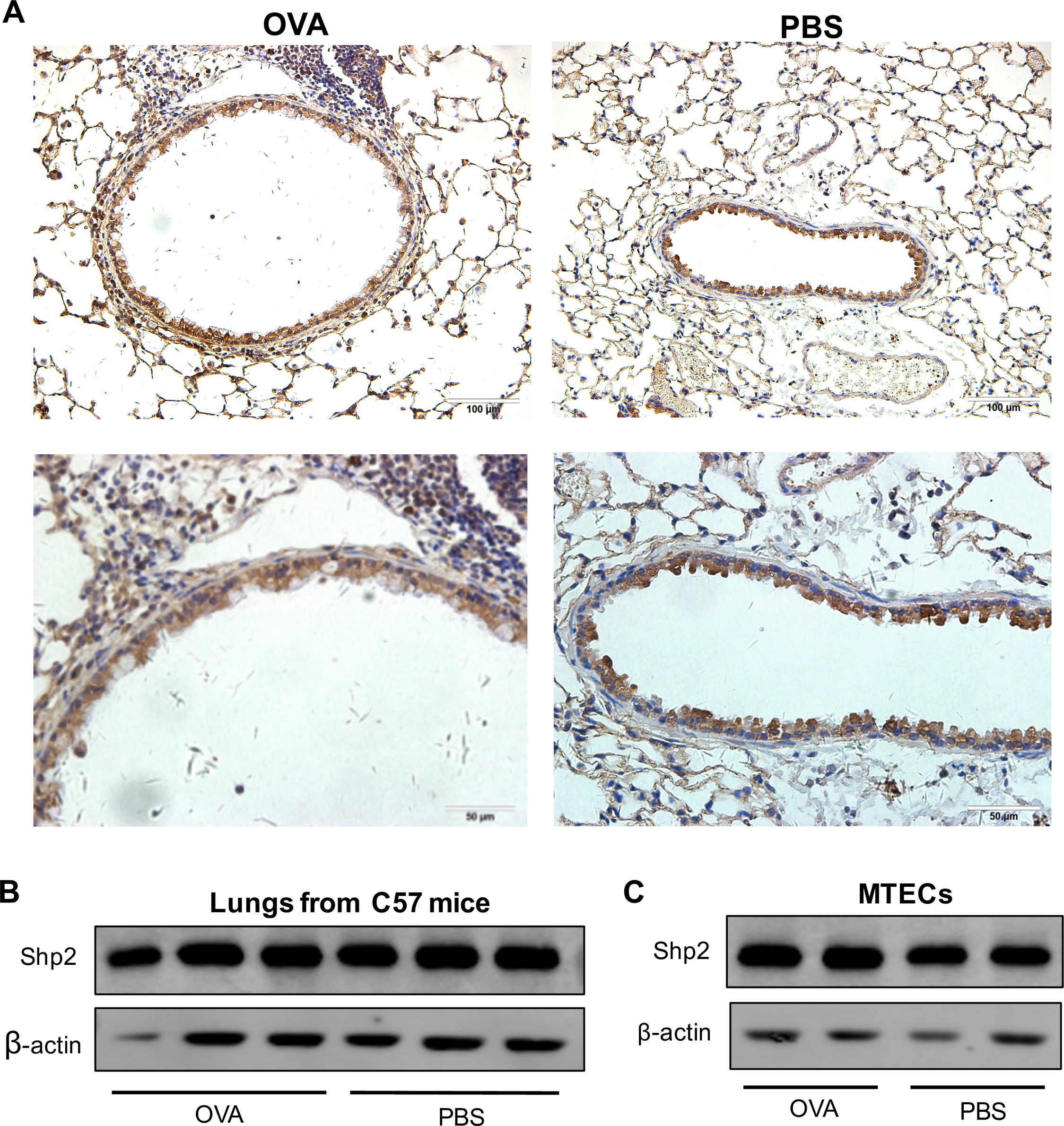

Supplement: S3 Fig — (A) The cellular distribution of Shp2 (brown staining cells) in the lungs of healthy and allergic mice by immunohistology. (B) Total Shp2 protein expression in the lungs of healthy and allergic mice by immunoblotting. (C) Shp2 protein expression in MTECs that respectively isolated from healthy and allergic mice (immunoblotting). (TIF) [file pone.0177334.s003.tif]

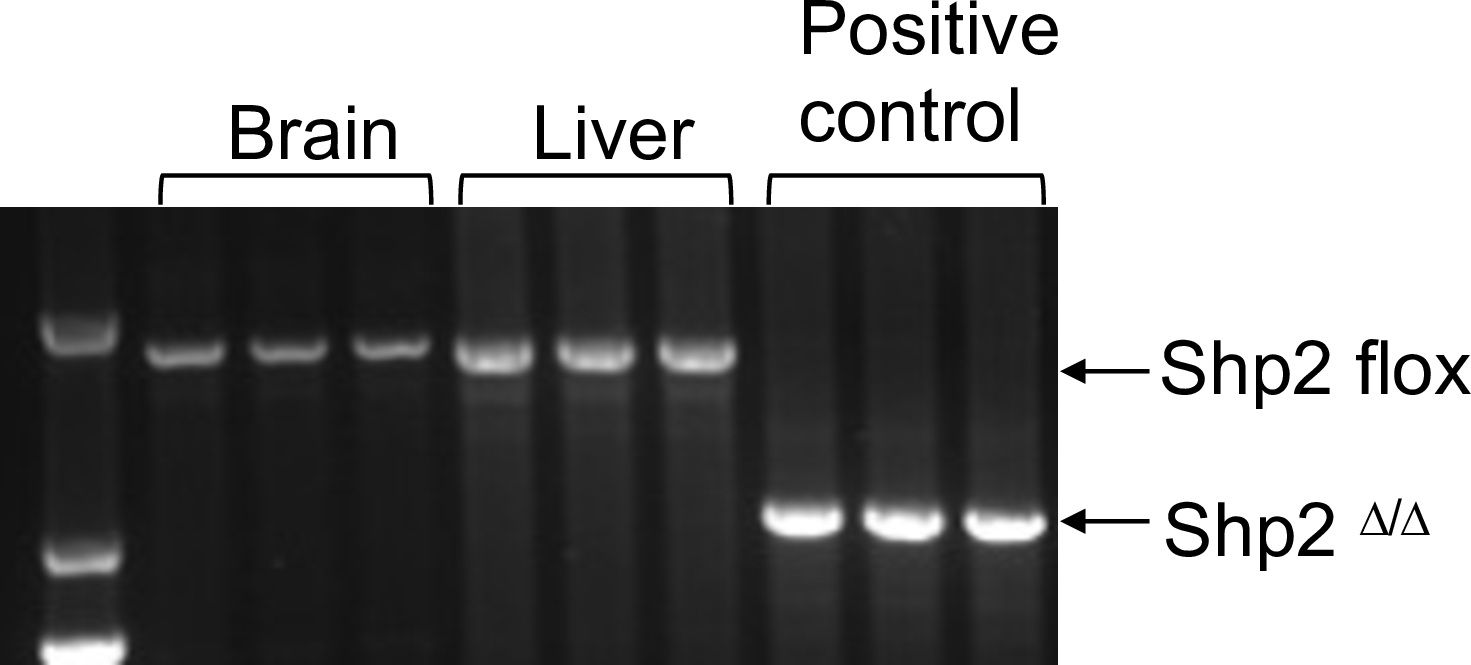

Supplement: S4 Fig — Genomic DNA was isolated from the brain and liver of CC10-rtTA/(tetO)7-Cre/Shp2f/f mice after 7-day treatment with DOX (through drinking, 2 mg/ml in H2O) and Shp2 allele was detected by PCR. (TIF) [file pone.0177334.s004.tif]
